# An Ab Initio Study of Pressure-Induced Reversal of Elastically Stiff and Soft Directions in YN and ScN and Its Effect in Nanocomposites Containing These Nitrides

**DOI:** 10.3390/nano8121049

**Published:** 2018-12-14

**Authors:** Martin Friák, Pavel Kroupa, David Holec, Mojmír Šob

**Affiliations:** 1 Institute of Physics of Materials, Academy of Sciences of the Czech Republic, Žižkova 22, CZ-616 62 Brno, Czech Republic; kroupavel@gmail.com (P.K.); mojmir@ipm.cz (M.Š.); 2 Department of Physics, Imperial College London, Prince Consort Road, London SW7 2BP, UK; 3 Department of Materials Science, Montanuniversität Leoben, Franz-Josef-Strasse 18, A-8700 Leoben, Austria; david.holec@unileoben.ac.at; 4 Department of Chemistry, Faculty of Science, Masaryk University, Kotlářská 2, CZ-611 37 Brno, Czech Republic; 5 Central European Institute of Technology, CEITEC MU, Masaryk University, Kamenice 5, CZ-625 00 Brno, Czech Republic

**Keywords:** YN, ScN, pressure, elasticity, ab initio, stability, nanocomposites

## Abstract

Using quantum-mechanical calculations of second- and third-order elastic constants for YN and ScN with the rock-salt (B1) structure, we predict that these materials change the fundamental type of their elastic anisotropy by rather moderate hydrostatic pressures of a few GPa. In particular, YN with its zero-pressure elastic anisotropy characterized by the Zener anisotropy ratio $A_{Z}=2C_{44}/\left(C_{11}-C_{12}\right)$ = 1.046 becomes elastically isotropic at the hydrostatic pressure of 1.2 GPa. The lowest values of the Young’s modulus (so-called soft directions) change from 〈100〉 (in the zero-pressure state) to the 〈111〉 directions (for pressures above 1.2 GPa). It means that the crystallographic orientations of stiffest (also called hard) elastic response and those of the softest one are reversed when comparing the zero-pressure state with that for pressures above the critical level. Qualitatively, the same type of reversal is predicted for ScN with the zero-pressure value of the Zener anisotropy factor $A_{Z}$ = 1.117 and the critical pressure of about 6.5 GPa. Our predictions are based on both second-order and third-order elastic constants determined for the zero-pressure state but the anisotropy change is then verified by explicit calculations of the second-order elastic constants for compressed states. Both materials are semiconductors in the whole range of studied pressures. Our phonon calculations further reveal that the change in the type of the elastic anisotropy has only a minor impact on the vibrational properties. Our simulations of biaxially strained states of YN demonstrate that a similar change in the elastic anisotropy can be achieved also under stress conditions appearing, for example, in coherently co-existing nanocomposites such as superlattices. Finally, after selecting ScN and PdN (both in B1 rock-salt structure) as a pair of suitable candidate materials for such a superlattice (due to the similarity of their lattice parameters), our calculations of such a coherent nanocomposite results again in a reversed elastic anisotropy (compared with the zero-pressure state of ScN).

## 1. Introduction

Anisotropic (tensorial) elastic characteristics belong to the most fundamental properties of crystals (see [1,2]) and reflect the nature of inter-atomic bonds. Elastic constants are decisive for numerous phenomena well beyond simple mechanical response of crystal lattices to uniaxial, biaxial or triaxial loading. In particular, strong long-range elastic interactions among point defects, such as substitutional or interstitial solute atoms, are inter-linked with their low solubilities (see [3]). Further, elastic interactions among extended defects, such as edge or screw dislocations [4], grain boundaries or stacking faults, are crucial for phenomena mediating plasticity in crystalline materials. As another example, when changing the temperature in composites, it is the varying lattice-parameter mismatch and elastic stiffnesses of coexisting phases which play an important role in stresses occurring at internal interfaces. Regarding the mechanical stability and the very existence of materials phases, relations among elastic constants are vital for the mechanical stability as violations of so-called Born-Huang stability criteria [5] often lead to phase transformations. As it would be indeed very difficult to provide a complete list of phenomena intertwined with elastic properties, we further mention only their role in sound propagation [6,7,8,9,10], heat transfer and partly also in thermal lattice vibrations [11], which significantly contribute to the thermodynamic stability (phonon entropy contribution is a part of the free energy [12]).

When considering elastic anisotropy of materials, its most important characteristics are the magnitude of the anisotropy (as a measure of how different the elastic response is from the isotropic one) and then special directions for which the studied crystalline system exhibits the softest and stiffest elastic response. Focusing on crystals with a cubic symmetry, which are described by three independent elastic constants (stiffnesses) $C_{11}$, $C_{12}$ and $C_{44}$, the magnitude of the elastic anisotropy is often expressed by the so-called Zener’s anisotropy ratio $A_{Z}=2C_{44}/\left(C_{11}-C_{12}\right)$. If this ratio exceeds one, the elastically stiffest response of the studied cubic crystal to uniaxial loading is found along the 〈111〉 family of directions while the softest response occurs along the 〈001〉 directions. When the Zener’s ratio is lower than one, the elastic anisotropy is opposite and the stiffest elastic response, i.e., the highest value of the Young’s modulus, is found along the 〈001〉 directions. The border case of $A_{Z}$ = 1 describes an elastically isotropic material (the directional dependence of Young’s modulus would be a sphere).

Together with the above discussed second-order elastic constants, there are also elastic constants related to higher-order elasticity [13,14,15,16]. In particular, there are six independent third-order elastic constants in the case of cubic-symmetry systems: $C_{111}=C_{222}=C_{333}$, $C_{144}=C_{255}=C_{366}$, $C_{112}=C_{223}=C_{133}=C_{113}=C_{122}=C_{233}$, $C_{155}=C_{244}=C_{344}=C_{166}=C_{266}=C_{355}$, $C_{123}$, $C_{456}$ and all other are zero (provided that the mutual orientation of the lattice and the coordination system is matching). Importantly, third-order elastic constants describe the changes of the second-order elastic constants due to the application of external stress or strain, including a hydrostatic pressure *p*.

In our study we show that the fundamental elastic anisotropy type, i.e., whether the elastically softest response is either along 〈001〉 or 〈111〉 directions and the corresponding Zener anisotropy ratio either higher or lower than 1, respectively, can be changed by application of moderate hydrostatic pressures. Importantly, the predicted reversal means that the mutual ratio of longitudinal sound velocities (which is faster or slower) in the [100] direction on one hand and in the [110] and [111] directions on the other, reverse as well [17]. Such a change has been reported mostly as a consequence of compositional changes so far, e.g., in Ref. [18], while we found that it caused by the hydrostatic pressure.

We predict the reversal for YN and ScN as two technologically important materials which have been intensively studied. Regarding YN, its electronic structure, vibrational spectrum, and thermal properties were computed using first-principles density functional theory (DFT) based simulations with a generalized gradient approximation (GGA) of the exchange correlation energy in [19]. The authors of that study also applied the Hubbard on-site correlation U term (GGA+U) and reported improvement in the accuracy of the calculation of the bandgap and selected features of the electronic structure of YN which are relevant to transport properties, such as transverse and longitudinal conduction band effective mass. The GGA+U calculations were also performed in the study of electronic, mechanical, and thermodynamic properties of YN in [20]. Other theoretical studies were focused on the stability of the rock-salt B1 structure of YN with respect to a pressure-induced transition into another phase, such as the caesium-chloride B2 one [21,22]. Full-potential calculations were also performed in the theoretical study by Stampfl and co-workers [23] who showed that local density approximation (LDA) predicts YN to be semimetal and the bandgap is open only when a screened-exchange calculations are performed. As far as ScN is concerned, it was a part of an extensive study of properties of 3d transition metal nitrides considering their cubic zinc-blende, rock-salt and caesium-chloride polymorphs [24]. It was also one of the compounds in the study of the Sc-based ternary nitrides [25] which was focused on single-crystal elastic constants, mechanical stability, the site-projected density of states, Fermi surfaces, charge densities and chemical bonding.

## 2. Methods

**Figure 1:**
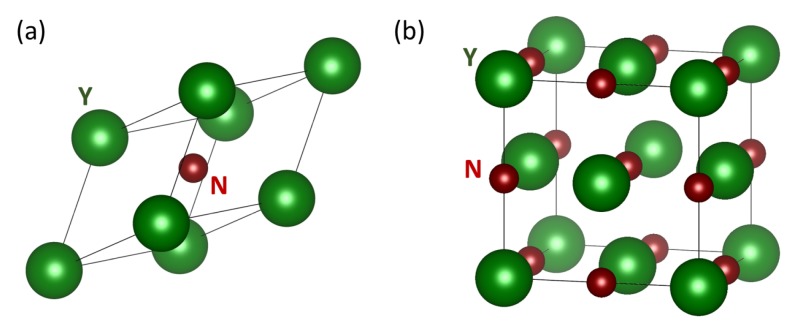
Schematic visualization of the 2-atom primitive (**a**) and 8-atom conventional cube-shape (**b**) unit cells of NaCl-structure of YN (some atoms are shown together with their periodic images).

Our quantum-mechanical calculations were performed within the framework of density functional theory [26,27] using the Vienna Ab initio Simulation Package (VASP) [28,29] and projector augmented wave (PAW) pseudopotentials [30,31] (electron configuration in the VASP-notation Y-sv: 4s4p5s4d, Sc-sv: 3p4s3d, N: s2p3). The computational setting was the same as in Ref. [32]. The exchange and correlation energy was treated in the generalized gradient approximation (GGA) as parametrized by Perdew and Wang [33]. We used a plane-wave energy cut-off of 800 eV, a 7 × 7 × 7 Monkhorst-Pack k-point mesh and 8-atom cube-shaped computational supercells (see a schematic visualization of this structure in Figure 1b). Second- and third-order elastic constants at zero pressure were computed as described in our paper [32] which also contains very detailed convergence tests. The second-order elastic constants under pressure (also in the case of tetragonal-symmetry states) were determined using the stress-strain method [34]. In this case, Born stability conditions in their original version are also valid for non-zero pressures and the external pressure does not enter here explicitly. In order to obtain highly accurate densities of states, 14 × 14 × 14 k-point meshes were used in the case of the above discussed 8-atom cells. These calculations were initially performed with employing the Fermi smearing (VASP-parameter ISMEAR = −1) with the smearing parameter σ = 0.02 eV. After reaching a self-consistent solution for a given geometry (for each studied lattice parameter), a non-selfconsistent run (VASP-parameter ICHARG = 11) was subsequently performed utilizing the tetrahedron method (VASP-parameter ISMEAR = −5) to compute the density of states (following the VASP manual). For phonon calculations we have used 64-atom 2 × 2 × 2 multiple of the cube-shape 8-atom elementary cell (which is shown in Figure 1b). The corresponding k-point mesh was then 4 × 4 × 4. Phonopy [35] software package was utilized.

## 3. Results

As far as the ground-state properties of B1-structure YN and ScN are concerned, the calculated equilibrium lattice parameters are in an excellent agreement with those previously obtained that employed different variants of the GGA exchange-correlation approximations as well as with experimental results. In particular, we find the lattice parameter of YN to be 4.916 Å when theoretical values 4.90–4.93 Å were reported in Reference [19], 4.619 Å in [20], 4.93 Å in [21,36], 4.85 Å in [23] and the experimental value is 4.88 Å [37]. Regarding ScN, our value 4.510 Å agrees with theoretical ones of 4.543 Å from [24], 4.516 Å reported in Reference [38], 4.50 Å in [23] and experimental 4.50 Å [39].

Regarding elastic properties, the first-order derivatives of the second-order elastic constants $C_{ij}$ with respect to the hydrostatic pressure *p* are in the case of cubic systems equal to (according to Refs. [13,14,15,32,40]):

(1) $$\frac{\delta C_{11}}{\delta p}=-\frac{2C_{11}+2C_{12}+C_{111}+2C_{112}}{C_{11}+2C_{12}},$$

(2) $$\frac{\delta C_{12}}{\delta p}=-\frac{-C_{11}-C_{12}+C_{123}+2C_{112}}{C_{11}+2C_{12}},$$

(3) $$\frac{\delta C_{44}}{\delta p}=-\frac{C_{11}+2C_{12}+C_{44}+C_{144}+2C_{166}}{C_{11}+2C_{12}}.$$

The calculated values of second-order $C_{ij}$(*p* = 0 GPa) and third-order elastic constants $C_{ijk}$(*p* = 0 GPa) determined for ground-state (i.e., zero hydrostatic pressure, *p* = 0 GPa) of YN and ScN are summarized in Table 1. The elastic constants were recently published in our previous work [32] but here we newly add also the changes δ*C*_11_/δp, δ*C*_12_/δp and δ*C*_44_/δp according to Equations (1)–(3). Regarding the calculated values of second-order elastic constants $C_{ij}$(*p* = 0 GPa), Table 1 shows that they are in an excellent agreement with previously published theoretical results for both YN and ScN when we selected GGA calculations [24,25,38] (LDA predicts both materials to be metallic [23]).

**Figure 2:**
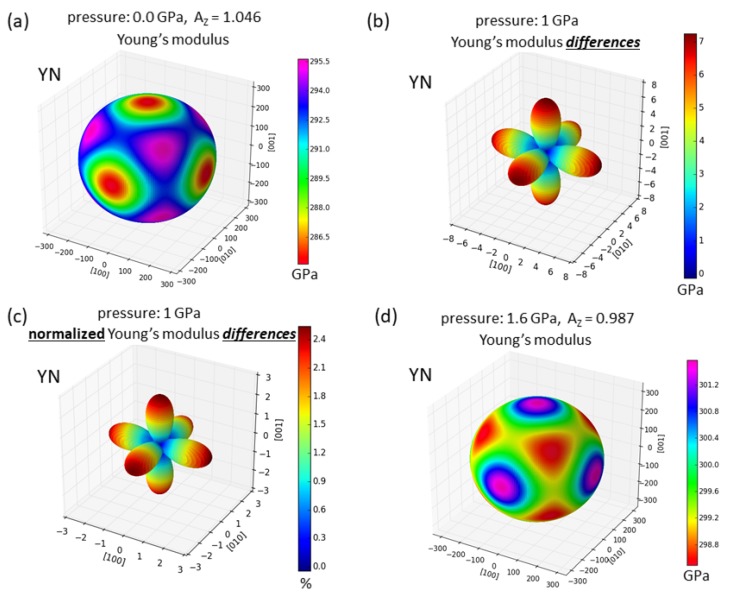
Computed changes in the elasticity of rock-salt structure YN visualized as directional dependencies of the Young’s modulus. The zero-pressure case based on the second-order elastic constants computed by the stress-strain method is shown in part (**a**). The estimated changes in the Young’s modulus due to 1 GPa of hydrostatic pressure are shown for different directions in absolute terms (in GPa) in part (**b**) and relatively (divided by the value for this direction in the zero-pressure case) in part (**c**). The visualized changes (in the second-order elasticity at the hydrostatic pressure of 1 GPa) are predicted using the second-order and third-order elastic constants computed for the zero-pressure state according to Equations (1)–(3). Finally, the directional dependence of the second-order elasticity computed at the 1.6 GPa is shown in part (**d**). Mind the change in the scale between the parts (**a**) and (**d**).

The second-order elastic constants of B1 structure YN for the zero-pressure case $C_{ij}$(*p* = 0 GPa) can be neatly visualized in the form of directional dependence of Young’s modulus *Y*(*p* = 0 GPa) in Figure 2a. The Young’ s modulus is a measure of the response of the studied system to an uniaxial loading along different directions and as such it reflects the elastic anisotropy. Young’s modulus in Figure 2a is nearly spherical, i.e., the elastic elastic anisotropy of YN at the zero-pressure case is weak and the corresponding Zener ratio $A_{Z}$ = 1.046. In order to graphically represent the third-order elastic constants $C_{ijk}$(*p* = 0 GPa), we conveniently visualize pressure-induced changes in the Young’s modulus for each direction. In particular, we show the difference between the Young’s modulus in the pressurized case *Y*(*p* = 1 GPa) and Young’s modulus for the zero-pressure case *Y*(*p* = 0 GPa), i.e., *Y*(*p* = 1 GPa) −*Y*(*p* = 0 GPa). The values of these changes (in GPa) are shown for each direction in Figure 2b while relative changes (when the difference *Y*(*p* = 1 GPa) −*Y*(*p* = 0 GPa) is for each direction divided by the *Y*(*p* = 0 GPa) along this direction) are visualized in Figure 2c.

Figure 2b,c show that the Young’s modulus is found to increase the most along the 〈001〉 directions. This is the direction along which the Young’s modulus of YN in the zero-pressure case exhibits the softest elastic response (the lowest value, see Figure 2a). In contrast, the change for the the 〈111〉 directions is nearly zero (see Figure 2b,c). Figure 2b,c thus indicates that application of hydrostatic pressure can change the type of elastic anisotropy.

The Young’s modulus of YN under pressure is predicted to have the stiffest (hard) elastic response along the 〈001〉 directions and not the softest one (as in the zero-pressure case). Such a change in the elastic anisotropy would be characterized by the change of the Zener anisotropy ratio which would become lower than that for pressurized states of YN. It is worth noting that these pressure-induced changes shown in Figure 2b,c are based on zero-pressure second- and third-order elastic constants. In order to check this prediction we have also determined the second-order elastic constants by quantum-mechanical calculations for a series of states at different hydrostatic pressures. Our results are shown in the form of directional dependence of Young’s modulus for the hydrostatic pressure of 1.6 GPa in Figure 2d. It can be seen that the softest elastic response (the lowest value of the Young’s modulus) is indeed along the 〈111〉 directions.

**Figure 3:**
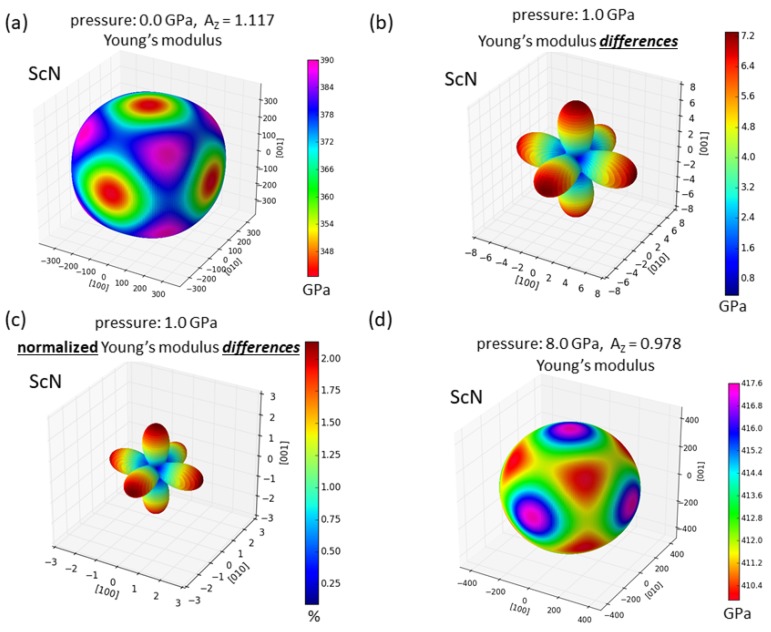
The same as in Figure 2 but for ScN. Part (**d**) is computed at the hydrostatic pressure of 8.0 GPa. The parts (**a**) and (**d**) were visualized by the SC-EMA [42,43,44] library (scema.mpie.de) based on our ab initio computed elastic constants.

While we expect that this reversal would be rather rare, we predict it also for ScN with the same rock-salt (B1) structure as in the YN case. Figure 3 visualizes the directional dependence of the Young’s modulus for ScN for the zero-pressure case (Figure 3a) as well as the impact of zero-pressure second- and third-order elastic constants on the elasticity of ScN (Figure 3b,c). Performing then calculations of second-order elastic constants also in the case of pressurized ScN (see Figure 3d), the comparison of Figure 3a,d clearly shows the change.

**Figure 4:**
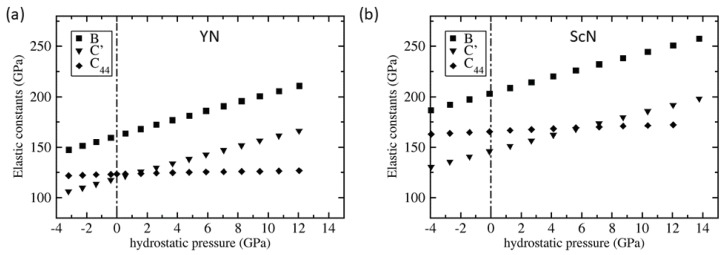
Quantum-mechanically calculated second-order elastic constants of YN (**a**) and ScN (**b**) for different hydrostatic pressures. The vertical dash-dotted lines indicate the zero hydrostatic pressure.

**Figure 5:**
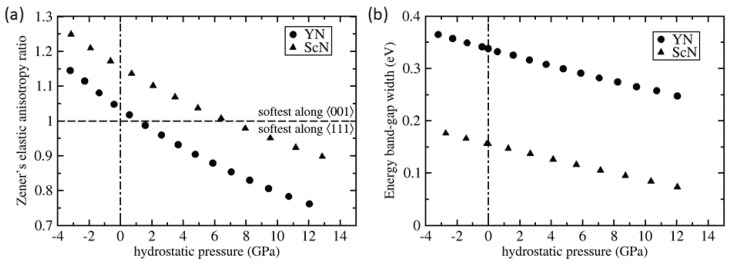
Quantum-mechanically computed (**a**) dependence of the Zener elastic anisotropy ratio $A_{Z}$ and the band-gap energy (**b**) as a function of hydrostatic pressure for both YN and ScN. The horizontal dashed line for $A_{Z}$ = 1 represents the border value of the elastic anisotropy, the vertical dash-dotted line corresponds to zero hydrostatic pressure.

In order to examine the changes in the elasticity in a broader range of pressures we have performed a series of calculations for YN and ScN states with different volumes (different hydrostatic pressures). The resulting elastic constants, the bulk modulus $B=(C_{11}+2C_{12})/3$, $C^{\prime }=\left(C_{11}-C_{12}\right)/2$ and $C_{44}$, are displayed in Figure 4. As the Zener’s ratio could be re-written as $A_{Z}=C_{44}/C^{\prime }$ the crossing of the trends for $C_{44}$ and $C^{\prime }$ indicates the change of the elastic anisotropy type. The pressure dependence of the Zener’s ratio is then depicted in Figure 5a. For YN the $A_{Z}$ ratio reaches the value of 1 (elastic isotropy) for the pressure of about 1.2 GPa. For higher pressures the type of elastic anisotropy is opposite to that in the zero-pressure state. The critical pressure for ScN is about 6.5 GPa (see Figure 5a).

Next, we have also checked the electronic properties of both materials which are predicted to be semiconductors in their ground state. This is in agreement with previous studies [19,20,23] but our value of the band-gap energy width (0.34 eV) is underestimated similarly as in previous theoretical studies in which similar computational methods were used—see a detailed discussion in Reference [20]. The pressure-dependences of the width of the energy band-gap in their electronic structures are depicted in Figure 5b. It is obvious that both YN and ScN remain semiconducting within the studied range of hydrostatic pressures.

The decreasing width of the energy band-gap with increasing (positive) hydrostatic pressure (lattice constants are smaller than the zero-pressure values) in both materials indicates that there can be a pressure-induced semiconductor-to-metal transition. Such a major change of the electronic structure (and subsequently properties of inter-atomic bonds) may also lead to a phase-instability and a transition into another crystal structure. For example, YN seems to be prone to a pressure-induced phase transition into the B2 (caesium chloride structure) phase according to full potential linearized augmented plane wave (FP-LAPW) calculations in [21,22], but at rather high pressures, 134 GPa [21] and 139 GPa [22]. As none of these transitions seems to be directly related to the change in the type of the elastic anisotropy reported in our present study, we do not examine them in detail and leave them for future work.

**Figure 6:**
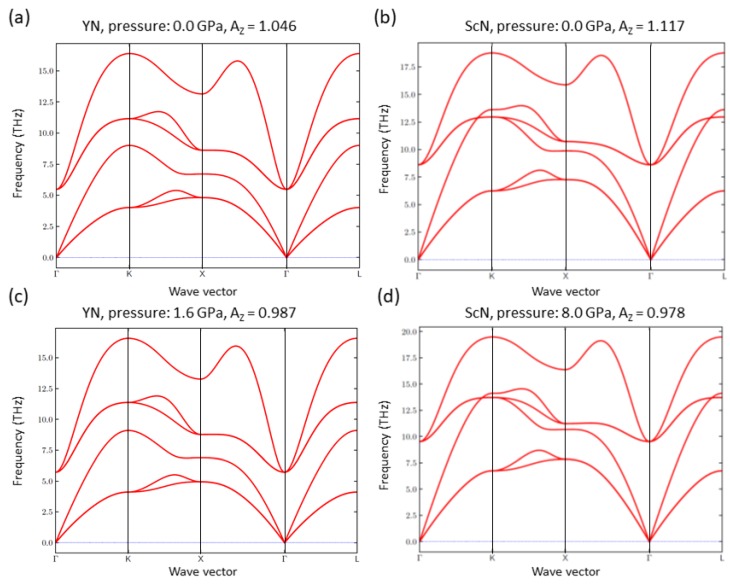
Quantum-mechanically calculated phonon dispersions at zero pressure for YN (**a**) and ScN (**b**) and for YN also for the hydrostatic pressure *p* = 1.6 GPa (**c**) and for ScN for *p* = 6.5 GPa (**d**).

As the elastic properties are inter-connected with phonon modes, we have also examined an impact of the above discussed change in the elastic anisotropy on vibrational properties. Our computed phonon spectra for both YN and ScN at the zero-pressure as well at selected pressures (for which the Young’s moduli are shown in Figure 2d and Figure 3d) are summarized in Figure 6. The change in the elastic anisotropy is found to have only a very minor impact on the vibrational properties.

**Figure 7:**
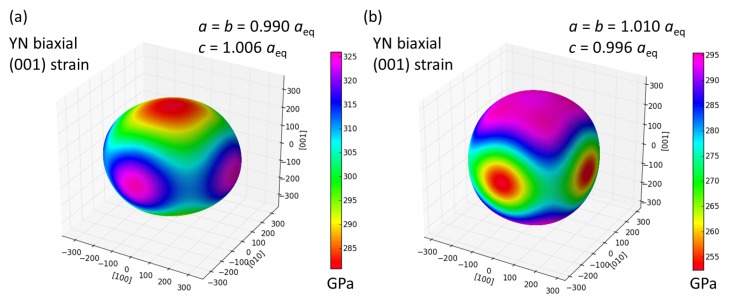
The calculated directional dependences of the Young’s modulus of two tetragonally deformed states of YN with $a=b=0.990\;a_{\mathrm{eq}}$ (**a**) $a=b=1.010\;a_{\mathrm{eq}}$ (**b**), respectively. The tetragonal lattice *c* parameters are equal to the values corresponding to the minimum energy (and zero stress $\sigma_{c}$ = 0).

After examining in detail the elastic-anisotropy change when applying hydrostatic pressures we next search for other conditions/mechanisms with potentially a similar impact. Our motivation is the fact that hydrostatic pressures over 1 GPa rarely occur in technologically relevant situations. It would be, therefore, desirable to achieve the studied elasticity change under more easily reachable conditions. It is interesting to examine biaxial loading conditions (misfit strains) which are induced, for example, in coherent nanocomposites (such as superlattices [45,46,47,48,49,50,51,52,53,54,55,56,57,58,59,60,61,62,63,64,65,66]) when materials with slightly mismatching lattice parameters co-exist. In order to simulate the impact of similar strain conditions, we have performed a series of calculations for tetragonally deformed YN. The YN cell then looses its cubic shape and symmetry and we conveniently describe it by two lattice parameters a=b and *c*. Considering the fact that these coherently-strained superlattices exist only in the case when the two co-existing materials have their lattice parameters only very slightly different (by about 1–2%), we limit our calculations to ±1.0% change of the lattice parameters with respect to the equilibrium lattice parameter (of the cubic-shape B1 lattice). Figure 7 shows the resulting directional dependencies of the Young’s modulus for biaxially 1% compressed case (Figure 7a) and biaxially 1% expanded state (Figure 7b), respectively. The tetragonal lattice parameters *c* of these states are equal to the values corresponding to the minimum energy (and zero stress $\sigma_{c}$ = 0) with the constraint that the lattice parameters a=b have those specific values.

The state with $a=b=0.990\;a_{\mathrm{eq}}$ and $c=1.006\;a_{\mathrm{eq}}$ (Figure 7a) is characterized by elastic constants $C_{11}=C_{22}=361$ GPa, $C_{33}=308$ GPa, $C_{12}=90$ GPa, $C_{13}=C_{23}=83$ GPa, $C_{44}=C_{55}=122$ GPa and $C_{66}=126$ GPa. The state with $a=b=1.010\;a_{\mathrm{eq}}$ and $c=0.996\;a_{\mathrm{eq}}$ (Figure 7b) has its elasticity described by elastic constants $C_{11}=C_{22}=283$ GPa, $C_{33}=325$ GPa, $C_{12}=75$ GPa, $C_{13}=C_{23}=79$ GPa, $C_{44}=C_{55}=123$ GPa and $C_{66}=120$ GPa.

Importantly, the reversal of the elastic anisotropy is clearly visible in Figure 7. Regarding the biaxially compressed state in Figure 7a, the highest value of the Young’s modulus is along the [±100] and [0±10] directions within the (001) plane of the biaxial loading. On the other hand, for the [00±1] directions from the {001} family, the lowest values of the Young’s modulus are found. As far as the biaxially expanded state in Figure 7b is concerned, the maximum Young’s modulus is along the [00±1] directions perpendicular to the (001) plane of the biaxial loading while the lowest values are obtained for the [±100] and [0±10] directions. The computed differences in the elastic response of the two biaxially loaded states indicate that tetragonal deformations may contribute to fine-tuning of elastic properties within a materials design of systems with a desired elasticity.

**Figure 8:**
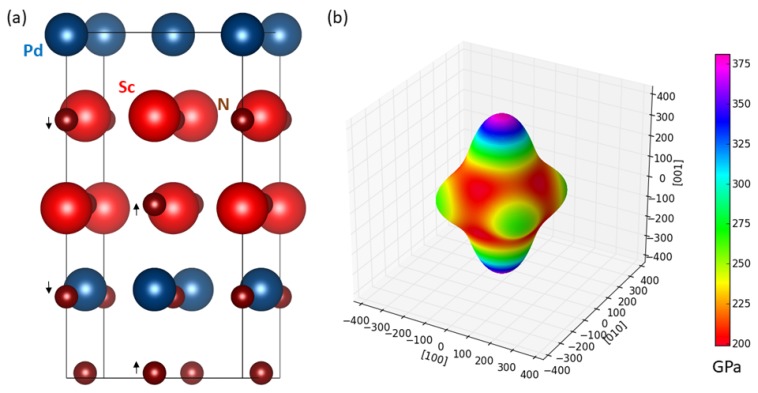
Schematic visualization of 16-atom ScN/PdN supercell (**a**) and the corresponding directional dependence of the Young’s modulus (**b**). The calculations for this nanocomposite were performed using 7 × 7 × 4 k-point grid. Small black arrows indicate the shifts of N atoms off the transition-metal planes perpendicular to the [001] direction.

Our previous simulations of tetragonally-deformed states of YN were motivated by strains appearing in coherent nanocomposites but, admittedly, a hypothetical partner material entered only via the geometry of the imposed strains. In order to check a more realistic situation with two different materials interfacing each other, we next simulate a superlattice consisting of two transition-metal (TM) nitrides each crystalizing in the B1 structure. As it has turned out, it is not easy to find another TM-nitride with the equilibrium lattice parameter close (±1–2%) to that of YN (our value: 4.916 Å). Therefore, we have tried to identify a partner material for ScN for which we found the lattice parameter equal to 4.510 Å. The motivation to replace the hydrostatic pressure of 6.5 GPa by another mechanism is even stronger in the case of ScN. Figure 8a visualizes a 16-atom supercell containing one conventional cell of ScN and one with PdN for which we obtained the lattice parameter equal to 4.447 Å (i.e., 1.4% smaller than in the ScN case). When applying periodic boundary conditions a coherent superlattice is formed. The composite supercell has a tetragonal shape and we will thus use the description by the lattice parameters a=b and *c* similarly as in the case of tetragonal states of YN discussed above. The calculated values are a=b = 4.532 Å and *c* = 8.690 Å. The former value means that ScN is biaxially expanded within the (001) plane by 0.49% and PdN is biaxially expanded by 1.91%. This unexpected results, expansions of both materials, is accompanied by contraction of both materials in the direction [001] perpendicular to the interfaces. In particular, the *c* lattice parameter of the composite is by 2.98% smaller than the sum of equilibrium lattice parameters of ScN and PdN. The reason for these unexpected results can be probably found in the fact that there is a structural distortion inside the composite. In particular, the N and TM atoms do not share the same planes which are perpendicular to the [001] direction (planes are parallel to the interfaces) as they do in the B1 ground-state structure. The off-sets are alternating (up/down) and their direction are schematically indicated by small arrows in Figure 8a. The magnitude of these shifts is (in relative terms as fractions of the supercell lattice parameter *c* and as absolute values) equal to ±0.0061 and ±0.053 Å in the PdN layers and ±0.0085 and ±0.074 Å in ScN layers. Similar shifts of N atoms were found also in MoN/TaN composites [47] and represent quite likely a frozen optical phonon mode.

Figure 8b then shows the elasticity of the studied ScN/PdN nanocomposite. The calculated elastic constants are $C_{11}=C_{22}=304$ GPa, $C_{33}=450$ GPa, $C_{12}=157$ GPa, $C_{13}=C_{23}=126$ GPa, $C_{44}=C_{55}=75$ GPa and $C_{66}=101$ GPa. When inspecting the directional dependence of the Young’s modulus in Figure 8b we can observe that the highest values are found for directions close to the [00±1] directions. Thus there is a way how a change of the elastic anisotropy can be achieved also for the ScN. But three aspects should be noted. First, the lowest value of the Young’s modulus is along the [±100] and [0±10] directions parallel to the plane of the composite interfaces (see Figure 8b). Second, the overall elastic anisotropy of ScN/PdN nanocomposite is an outcome of complex interactions of pre-strained materials each having a different tensorial elastic properties. Finally, the single-phase PdN in the B1 lattice has, according to our calculations, the Zener’s ratio equal to 0.631, i.e., opposite to that of single-phase B1-structure ScN. Therefore, a reduction of the Zener’s ratio due to the presence of PdN in the nanocomposite is then expected. Nanocomposites formed by either YN (or ScN) on one hand and other materials on the other require a detailed investigation and will be a topic of future studies.

## 4. Conclusions

We have performed a series of quantum-mechanical calculations of second- and third-order elastic constants of YN and ScN with the rock-salt structure in the case of their zero-pressure states as well as for systems compressed by hydrostatic pressures. We predict that both YN and ScN undergo a reversal of their elastic anisotropy type. In particular, their elastic anisotropy expressed by the Zener ratio drops under one due to applied hydrostatic pressure. At zero pressure, both systems exhibit the softest elastic response to uniaxial loading (the lowest value of the Young’s modulus) along the 〈100〉 directions which is changed to the 〈111〉 directions for pressures beyond the critical one. These transition pressures are rather moderate, 1.2 GPa and 6.5 GPa for YN and ScN, respectively. The elasticity change keeps the semiconducting character of both materials and has only a minor impact on the vibrational properties. As alternative mechanisms leading to the reversal of the elastically soft and hard directions, we identified tetragonal deformations of YN for very small biaxial strains (lattice parameter compressed/expanded by about 1%) and a composite (superlattice) state of ScN and PdN (mismatch of their lattice parameters is 1.4%). The last two discussed mechanisms clearly pave a path towards a strain-controlled fine-tuning of elastic anisotropy in materials, which would allow, for example, a theory-guided design of nanocomposites with a particular ratio of longitudinal sound velocities in the [001], [011] and [111] directions in different components.

## Figures and Tables

**Table 1:** Calculated second-order elastic constants $C_{ij}$(*p* = 0 GPa) (in comparison with selected literature values—when a GGA was used as in our case) and their pressure changes (δ*C*_11_/δp, δ*C*_12_/δp, and δ*C*_44_/δp) as approximatively evaluated for δp = 1 GPa from computed third-order elastic constants $C_{ijk}$(*p* = 0 GPa). Theoretical values taken from Ref. [24] are related to GGA-PW91 approximation [33] similarly as in our case (marked by *), GGA-PW91 + U (marked by **), GGA-PBE [41] (marked by ^†^) or GGA-PBE + U (marked by ^††^).

	*C*_11_	*C*_12_	*C*_44_	δ*C*_11_/δp	δ*C*_12_/δp	δ*C*_44_/δp
YN	318	81	124	7.55	1.12	−0.70
	(321 [24] *)	(81 [24] *)	(124 [24] *)			
	(304 [24] **)	(76 [24] **)	(122 [24] **)			
	(317 [24] ${}^{†}$)	(80 [24] ${}^{†}$)	(123 [24] ${}^{†}$)			
	(310 [24] ${}^{††}$)	(81 [24] ${}^{††}$)	(124 [24] ${}^{††}$)			
ScN	388	106	166	7.49	1.02	−0.51
	(399 [24])	(96 [24])	(158 [24])			
	(397 [25])	(131 [25])	(170 [25])			
	(354 [38])	(100 [38])	(170 [38])			
	*C* ${}_{111}$	*C* ${}_{112}$	*C* ${}_{123}$	*C* ${}_{144}$	*C* ${}_{166}$	*C* ${}_{456}$
YN	−4100	−160	180	180	−225	185
ScN	−5100	−190	260	200	−330	215
